# Thin Film Encapsulation for RF MEMS in 5G and Modern Telecommunication Systems

**DOI:** 10.3390/s20072133

**Published:** 2020-04-10

**Authors:** Anna Persano, Fabio Quaranta, Antonietta Taurino, Pietro Aleardo Siciliano, Jacopo Iannacci

**Affiliations:** 1 IMM-CNR, Institute for Microelectronics and Microsystems, National Research Council, Via Monteroni, 73100 Lecce, Italy; fabio.quaranta@cnr.it (F.Q.); antonietta.taurino@le.imm.cnr.it (A.T.); pietro.siciliano@le.imm.cnr.it (P.A.S.); 2 CMM-FBK, Center for Materials and Microsystems, Fondazione Bruno Kessler, Via Sommarive 18, 38123 Povo – Trento, Italy; iannacci@fbk.eu

**Keywords:** 5G, RF MEMS, thin film encapsulation, silicon nitride, oxygen plasma etching

## Abstract

In this work, SiN_x_/a-Si/SiN_x_ caps on conductive coplanar waveguides (CPWs) are proposed for thin film encapsulation of radio-frequency microelectromechanical systems (RF MEMS), in view of the application of these devices in fifth generation (5G) and modern telecommunication systems. Simplification and cost reduction of the fabrication process were obtained, using two etching processes in the same barrel chamber to create a matrix of holes through the capping layer and to remove the sacrificial layer under the cap. Encapsulating layers with etch holes of different size and density were fabricated to evaluate the removal of the sacrificial layer as a function of the percentage of the cap perforated area. Barrel etching process parameters also varied. Finally, a full three-dimensional finite element method-based simulation model was developed to predict the impact of fabricated thin film encapsulating caps on RF performance of CPWs.

## 1. Introduction

Radio-frequency microelectromechanical systems (RF MEMS) technology is emerging as a key enabling solution to address the demanding requirements that upcoming fifth generation (5G) standards pose upon passive devices and networks, such as high operating frequencies, large tunability, reduced hardware redundancy, and low power consumption [1,2,3]. These performance benefits, however, are offset by a lack of low-cost packaging available for RF MEMS. The package must protect the device from structural damage and contaminants, introduce minimal RF losses, and not degrade the performance of the switch, circuit or complex passive device. To enable widespread implementation of RF MEMS, the package ideally should also be low cost, require little additional space on the wafer, and be easy to incorporate in microwave integrated circuits.

MEMS switches can be packaged using wafer-level techniques, in order to avoid the high costs and possible damages due to individual handling and release. Wafer-level bonding and thin film encapsulation are the most common wafer-level packaging methods. In the first case, a ring is applied around the switch and a capping wafer is sealed by solder, glass frit, or friction [4,5]. The bonding ring causes a significant increase in the device footprint, and the capping wafer may create a high aspect ratio device. Metal-based sealing with solder or Au friction bonding is performed at low temperatures with the possible introduction of high RF losses. Glass-based sealing adds minimal RF loss, but typically requires high temperatures that may damage the switch.

By comparison, thin film encapsulation schemes offer a low profile and wafer-level integrated-circuit compatibility. Thin film encapsulation typically uses a sacrificial layer to cover the structures to be sealed, followed by a cap film deposition. The sacrificial layer is subsequently removed by etching through access holes or by thermal decomposition. Finally, a sealing film is deposited over the cap film to seal the access holes.

The choice of the material for the encapsulating film is an important concern. With this regard, several materials (organic, metals, and dielectrics) have been proposed [6,7,8,9,10,11,12] with thicknesses in the range of 1–20 µm. Among the various materials, aluminum nitride, amorphous silicon, and silicon nitride are the most used [7,9,10,11,12] for thin encapsulating layers. In fact, they satisfy some essential requirements to make the packaging process suitable for industrial production, these include: a good structural integrity and selectivity with respect to the sacrificial material during the chemical etching; an excellent insulation during device operation; and an optimized deposition rate in relation to the thickness to achieve. In particular, mixed frequency plasma-enhanced chemical vapor deposition (PECVD) provides low stress silicon nitride layers that are stable if processed at high temperature [13].

Another crucial issue of thin film encapsulation is that the etch times required to remove the sacrificial material can be excessively long, especially as the size of encapsulation becomes larger, limiting the manufacturability of the package. The long release time may limit the choices of cap and sacrificial layer materials, due to the possible damage of cap layer and/or encapsulated device during the release process. Etch holes distributed over the cap layer [10,12] or fabricated in its sidewall [12] with standard surface-micromachining techniques can alleviate this problem. Anisotropic plasma assisted etching processes, based on fluorine chemistry, are generally used in the reactive ion etching (RIE) or inductively coupled plasma (ICP) configurations to pattern the encapsulation caps with etch holes. The drawback of RIE and ICP methods is that the substrate bias and physical ion bombardment can modify the stress distribution in encapsulated suspended beams, due to device heating with the consequent beam deformation [14]. 

Inorganic materials, like silicon oxide and amorphous silicon, are generally used as sacrificial materials [11,12], but their etching requires F-based vapors that could be a serious challenge in combination with micromachining technologies for structures containing aluminum or silicon oxide. Organic materials, which can be easily removed with oxygen plasma etching, are interesting candidates for sacrificial layers [7,10] in fabrication processes at low temperature (<200 °C).

In this work, encapsulating caps were fabricated on conductive coplanar waveguides (CPWs), in view of their application for packaging of RF MEMS in fifth generation (5G) and modern telecommunication systems. Capping layers in the thin multilayer SiN_x_/aSi/SiN_x_ were deposited by a mixed frequency PECVD. The technological novelty aspect of this work is that a barrel etching was used to pattern the capping layers as an alternative to RIE and ICP processes. This allows an entirely chemical process with no ion bombardment to be applied to the device. Moreover, the use of the barrel etcher for both the pattern and release of the capping layer offers the advantage of reducing fabrication costs; since the encapsulating layer is sequentially etched and released in the same chamber, the plasma chemistry only needs to be changed from fluorine to oxygen-based chemistry. The removal of the sacrificial layer was investigated as a function of the percentage of cap perforated area as well as the time (t) and the power (P) of the barrel etching. Finally, a full three-dimensional (3D) finite element method-based (FEM) simulation model was used to predict the RF performance of uncapped and capped CPWs. 

## 2. Outlook of 5G and RF Passives Requirements

Across the last four decades, there has been a seamless spread and evolution of mobile communication devices and services, starting from the first cellular network generation (1G) during the 1970s and 1980s, to the current fourth generation Long Term Evolution (4G-LTE), deployed in 2010 [15,16,17,18,19,20,21,22]. Given this scenario, the upcoming 5G of mobile communications will step forward along the visionary direction of an all-communicating world [23,24]. Such a perspective can be inflected in high-level specifications according to the following reference list [25,26,27,28]:

(1) Increase of data volume up to 1000 times;
(2) Increase of connected devices from 10 to 100 times;
(3) Typical user data rate increased from 10 to 100 times;
(4) Extended battery life up to 10 times;
(5) End-to-End (E2E) latency down to the milliseconds range.

The 5G will have to realize a common connected platform able to satisfy the above-listed specifications. This will require acting at diverse technical levels [29,30,31,32,33]:

(a) Radio links;
(b) Multi-node/multi-antenna transmission;
(c) Network dimension;
(d) Spectrum usage.

Given the system-level specifications discussed above, it is now necessary to frame how they reflect in terms of characteristics and requirements that basic hardware components and RF passives must comply with, in order to sustain and empower the high-level vision of 5G. To this purpose, as the first step it is appropriate to list the classes of RF passive components and networks, which will be necessary for 5G applications. It must also be stressed that all the devices mentioned can be effectively realized in RF MEMS technology [34]:

(1) Wideband switches and switching units with low-loss, high-isolation, very-low adjacent channels cross-talk, working from 2–3 GHz up to 60–70 GHz (and more);
(2) Reconfigurable filters with high stopband rejection and low attenuation of the passed band;
(3) Wideband multi-state impedance matching tuners;
(4) Programmable digital step attenuators with multiple configurations and flat characteristics over 60–70 GHz frequency intervals;
(5) Wideband multi-state/analogue phase shifters;
(6) Hybrid devices with phase shifting and programmable attenuation (i.e., examples 4 and 5 as a unique device);
(7) Miniaturized antennas and arrays of antennas integrated monolithically with one or more of the previous devices.

Now that the main macro-classes of RF MEMS passives that 5G will demand are mentioned, it is useful to list the most relevant tentative specifications that such devices will have to meet in order to be successfully employed within 5G-related applications. Such specifications are briefly listed as follows:

- Frequency range: From sub-6 GHz up to mm-wave range (60–70 GHz and more);
- Isolation: Better than −30/−40 dB for frequencies as high as possible;
- Loss: Below –1 dB on the widest possible frequency range;
- Cross-talk: Below −50/−60 dB over the widest frequency range possible;
- Switching time: Lower than 1 ms, with few fractions of μs (e.g., 200–300 μs) as reasonable target;
- Control voltage: Within a few Volts (e.g., 2–3 V).

In light of the just sketched scenario, RF MEMS technology has already demonstrated its ability to address the demanding requirements imposed by 5G on RF passive components, in terms of wide reconfigurability/tunability, wideband operability, and frequency agility [1,2,35]. Nonetheless, a crucial aspect to be carefully handled, in order to exploit the full commercial potentialities of RF MEMS, is that of packaging and integration.

As a matter of fact, microsystems technology is either incompatible or difficult to blend monolithically with standard semiconductor technologies (e.g., CMOS). As a result, employment of RF MEMS components within systems and sub-systems always leverages integration via surface mount technologies (SMTs), like flip-chip, ball grid arrays (BGAs), wire-bonding, and so on [36]. In addition to these aspects, MEMS devices in general need to be encapsulated and protected. In fact, microsystems need a package due to their fragility and exposure to harmful environmental factors, including mechanical shocks, contamination, presence of dust particles, moisture, etc. [37].

When dealing with RF MEMS, additional aspects to be carefully tackled come into the equation. The application of a protective cap to RF passives means introducing various parasitic effects that can detune the electromagnetic performance of the devices and cause additional loss [38]. More in detail, applying a protective package to RF MEMS means building a housing for the device with a physical part (i.e., a dome) in the vicinity of the RF passive itself. Such a dome, therefore, will interact with the electromagnetic field around the device, thus introducing additional and unwanted capacitive couplings. Moreover, very often the packaging solution requires managing the redistribution of the electrical signals from the RF MEMS device to the external world. This can be done, for instance, by means of through-silicon vertical vias filled with conductive materials, or by means of signal underpasses [39,40]. Regardless of the specific signal redistribution solution, additional resistive losses, discontinuities of the RF paths, series inductive, and shunt capacitive contributions are added, thus jeopardizing the characteristics of the initial RF MEMS device. This is the reason why the electromagnetic design of the package should be optimized with at least the same care spent for optimizing RF MEMS devices themselves [41,42].

The packaging strategy proposed in this work and discussed in the following pages, relies on the thin film capping approach. This means that no additional paths for the RF signals are needed. However, the interaction of the capping dome can still represent a problem. This is the reason why electromagnetic simulations will be reported and discussed in the following sections.

Our investigations at this stage refer to simple CPWs. This is because of a two-fold motivation. On one side, CPWs are easier and faster to fabricate as test structures for the subsequent application of the protective package. On the other hand, RF MEMS are always framed within CPW structures. Therefore, apart from switching and reconfigurability functions, the electromagnetic behavior of RF MEMS is very similar to that of standard CPWs.

## 3. Fabrication Flow Chart

**Figure 1:**
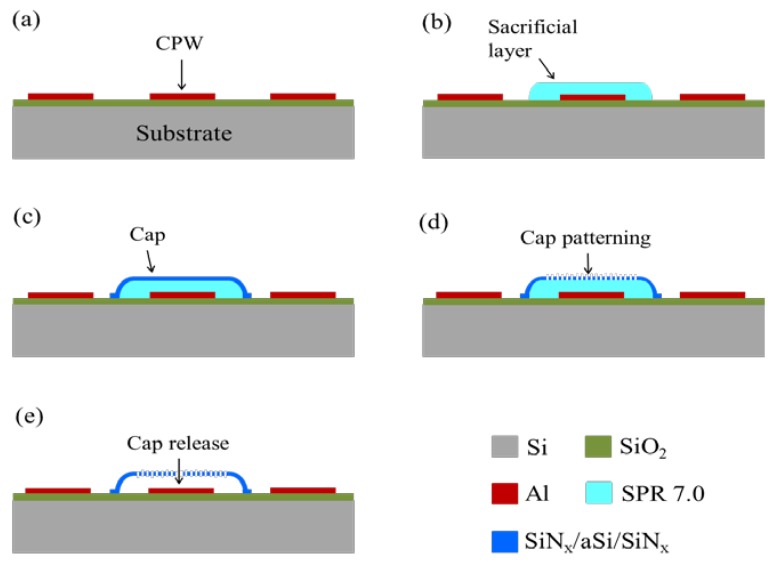
Sketch of the process flow for the fabrication of thin film encapsulating caps.

Figure 1 shows a sketch of the process flow for the fabrication of the thin film encapsulating caps. All samples were fabricated on 3 in, ISO standard silicon wafers, covered with 500-nm thick silicon oxide. A 450-nm thick aluminum layer was deposited at room temperature by RF sputtering and patterned by optical lithography and lift-off techniques to define CPWs (Figure 1a). The positive resist SPR7.0 was used with a thickness of 8.5 μm as the sacrificial layer to define the air gap areas under caps on the central conductor of CPWs (Figure 1b). Thin films in the trilayer SiN_x_/a-Si/SiN_x_ with thicknesses of 220/350/227 nm respectively, were deposited in a single process using a coupled planar parallel electrode Multiplex Series PECVD (Surface Technology Systems, Ltd) with an electrode diameter of 24 cm (Figure 1c). The system was equipped with a high frequency (HF) generator at 13.56 MHz and a low frequency (LF) generator at 380 kHz. The operating temperature of the shower head of the machine was 200 °C, while the temperature of the chuck where the wafer was placed during the deposition process was 150 °C. The other parameters for nitride depositions were: 40 sccm of NH_3_, 40 sccm of SiH_4_, 1960 sccm of N_2_, chamber pressure of 900 mTorr, HF generator power of 30 W, LF generator power of 20 W, HF/LF cycle time of 8 s/2 s. The parameters for the deposition of a-Si were: 20 sccm of SiH_4_, 1000 sccm of Ar_2_, chamber pressure of 1000 mTorr, LF generator power of 50 W, process time of 9 min. 

Stress measurements, using the Stoney wafer curvature variation equation [43], were performed with a profilometer KLA-Tencor P6 surface profiler, equipped with a 2-µm diamond stylus tip. Stress measurements were carried out on layers of SiN_x_, a-Si, and SiN_x_/a-Si/SiN_x_ directly deposited on the wafer in order to achieve an indication of the stress inside the suspended caps that will be fabricated with these material layers. Tensile stress values of 14 MPa, 12.6 MPa, and 24 MPa were measured for SiN_x_, a-Si, and SiN_x_/a-Si/SiN_x_ layers, respectively. The stress measurement for the trilayer is greater than the expected value, which is the weighted volumetric average of the single layers. A possible reason for this result is that the first nitride layer undergoes an increase of the residual stress during the depositions of the amorphous silicon and the second nitride layers, due to the heating and subsequent H outgassing [44]. Three profiles were measured for each sample along its diameter; the resulting stress data show a standard deviation better than 5%. 

Two barrel etchings were performed in the same chamber in order to create a matrix of holes through the SiN_x_/a-Si/SiN_x_ caps (Figure 1d) and to remove the sacrificial layer (Figure 1e). The barrel parameters used for the two etching processes are summarized in Table 1. For the patterning of top and bottom SiN_x_ layers, an etching time of 60 s and 90 s was used, respectively. Power and time of the barrel process for cap release were varied, as discussed in detail in Section 5. 

Matrixes containing a different number of holes with a diameter (D) of 7 and 8 µm were etched through the caps, resulting in a different percentage of perforated area, as summarized in Table 2. 

**Figure 2:**
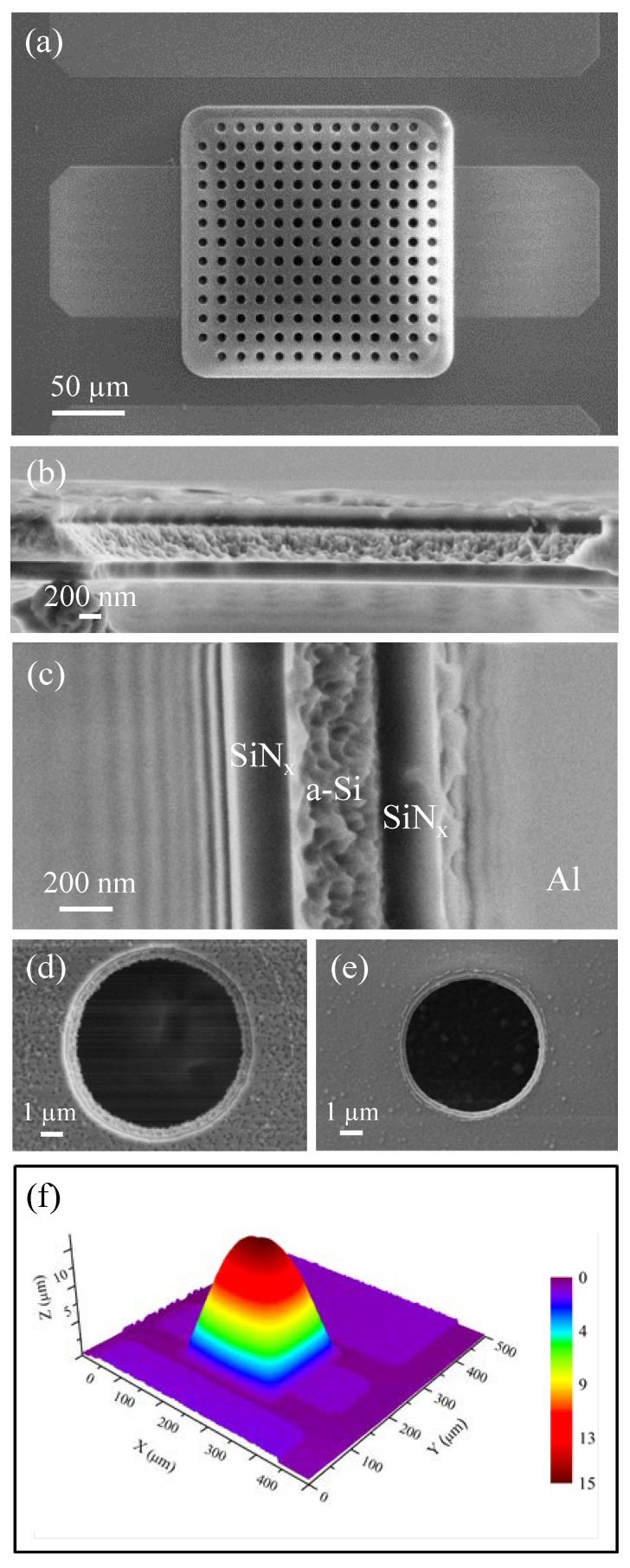
Scanning electron microscopy (SEM) view of a fabricated encapsulating cap (**a**). SEM cross-section of the SiN_x_/a-Si/SiN_x_ trilayer at lower (**b**) and higher magnification (**c**), along a cleaved hole. SEM view of a hole through the cap surface after barrel etching (**d**) and inductively coupled plasma (ICP) etching (**e**). 3D surface profile of the cap in (**a**) under the application of a stylus tip force of 4.9 µN (**f**).

Caps with a size of 200 µm × 200 µm were fabricated on the central conductor of CPWs (Figure 2a). The morphology of the SiN_x_/a-Si/SiN_x_ trilayer and the relevant interfaces were observed by scanning electron microscopy (SEM) in cross-sectional geometry, as shown in Figure 2b,c. The micrographs show the section of a cleaved hole, as clearly visible in the low magnification image in Figure 2b; therefore, the layers’ morphology is the native one (i.e., resulting from the etching process), and is not affected by the cleavage procedure. It is worth noting that the hole sidewalls are not vertical, and this is particularly evident for the a-Si film, forming a surface at about 50 degrees with the hole plane and exhibiting a very rough morphology. This morphology is probably the result of the granular structure of the a-Si film, while the SiN_x_ layers appear compact and homogeneous (Figure 2c). Thickness uniformity of layers deposited on the Si wafer was checked and found homogeneous below 5%. Figure 2d shows a typical SEM view of a hole with a diameter of 7 µm, which was obtained in the barrel etcher. For comparison, the same hole obtained changing the barrel etcher with an ICP etcher is shown in Figure 2e. It is notable that the trilayer structure of the capping layer is more evident in the case of the hole etched in the barrel reactor, due to the inclined sidewalls, as already observed in the cross sectional analysis. Furthermore, the diameter of the barrel hole is about 30% larger than the ICP hole. Both these effects are due to the isotropic characteristic of the barrel etching. Fabricated caps show a concave shape with a height of ~15 µm (Figure 2f). 

## 4. Release as a Function of Cap Holes

**Figure 3:**
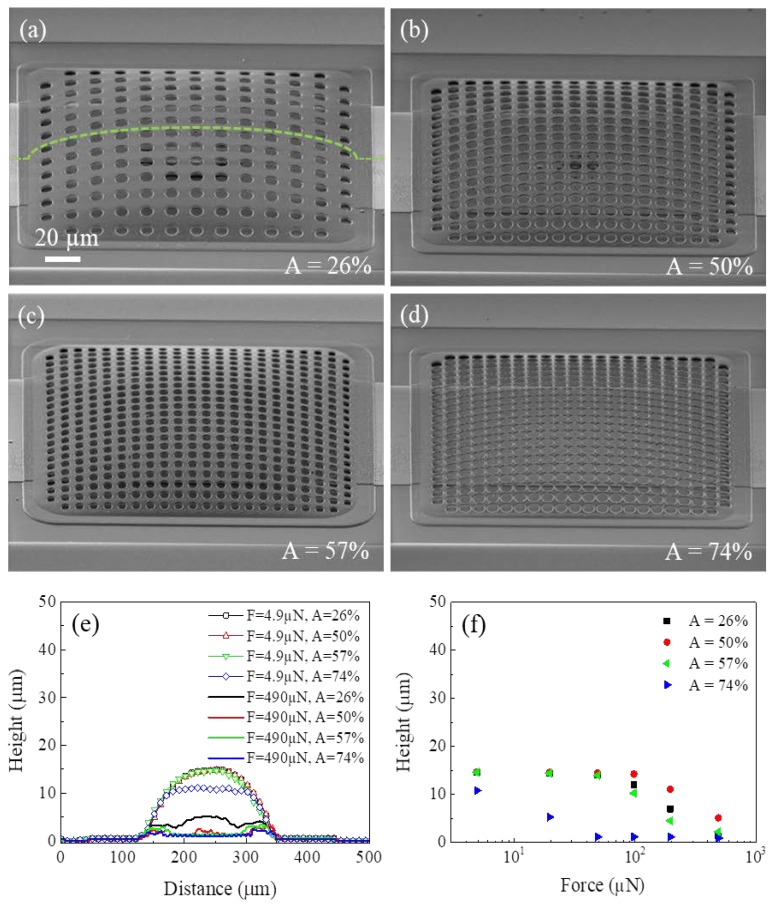
Tilted SEM images (tilt angle of 60°) of the fabricated encapsulating caps with A = 26% (**a**), 50% (**b**), 57% (**c**), and 74% (**d**). Profiles measured for caps in (**a**–**d**) under the application of a stylus tip force of 4.9 µN and 490 µN (**e**). Deflection in the center of the caps in (**a**–**d**) as a function of the stylus tip force (**f**). The marker is the same for all SEM images. The green line in (a) is the profiler scan line. Barrel parameters, which were used for the release of caps in (**a**–**d**), are P (power) = 600 W and t = 8 min.

Surface profilometry and SEM investigations were combined to evaluate the removal of the sacrificial layer as a function of the percentage of cap perforated area as well as the mechanical robustness of the fabricated caps. Figure 3a–d shows caps with different hole matrixes, which were released after the same barrel etching with t = 8 min and P = 400 W. For A = 26%, a residual sacrificial layer was observed under the central part of the cap (Figure 3a). By increasing A up to 50%, the residual sacrificial layer reduces, but does not disappear (Figure 3b). The complete release of the cap is observed at A = 57% (Figure 3c) and A = 74% (Figure 3d); the highest A value corresponds to the largest perforated area that can be etched without the cap collapsing on the CPW. SEM analysis was performed for caps released with different powers and times of the barrel etching. It was found that similar results were obtained for t = 8 min, whereas the sacrificial layer was completely removed even for A = 26% when t = 20 min and P > 300 W or t = 80 min and P > 100 W were used.

Linear profiles along the caps in Figure 3a–d were performed under the application of different stylus tip forces. Figure 3e shows the comparison between the scans along caps under the application of the lowest (4.9 µN) and the highest (490 µN) force values. In the case of the lowest force, the profile is approximately the same for caps with A < 57%, while a lowering and a flattening are observed for the cap with A = 74%. When the highest force value of 490 µN is applied, the caps tend to collapse on the CPW; that is, they bend up to touch the CPW, with possible evidence of residual sacrificial material. Consistent with SEM images, the collapse of the cap on the CPW is not complete for A = 26% and A = 50% since a feature of a few microns was observed in the central part of the cap. The height of this feature reduced from 5 µm to 2 µm as A increased from 26% to 50%. For greater values of A, the cap was completely collapsed on the central conductor of the CPW with no evidence of residual sacrificial material. In all cases, the cap results were stiffer at the anchoring points than in the internal region, as already observed in another study [10]. Figure 3f shows how the center of caps with the various A values deflects as a function of the stylus tip force. It is observable that the cap with A = 26% shows a deflection for forces greater than 100 µN. In particular, it deflects by ~30% under the application of 196 µN and even more when the applied force is increased, until the maximum deflection for F = 490 µN when the cap collapsed on the residual sacrificial material. With increasing A from 26% to 50%, the cap bends for F = 98 µN with a deflection of 2 µm. Flexibility further increases for the cap with A = 57%. The cap with A = 74% collapses on the CPW for a force as low as 49 µN, however no breakage is observed for forces up to 490 µN. 

## 5. Release as a Function of Barrel Parameters

Power (P) and time (t) of the barrel etching, which was used for cap release, were varied in the ranges 100–600 W and 8–80 min, respectively. The aim of these tests was to identify the right trade-off between power and time, in order to achieve the complete release of the cap without any damage. The different barrel etching parameters and the results in terms of cap release are summarized in Table 3. 

It is notable that with the powers of 600 and 400 W, the complete cap release was achieved with an etching time of 8 min. By reducing the power to 300 W, 8 min were not enough to achieve the complete release that was, instead, obtained at 20 min. We also tested what happens if an etching time longer than 20 min is used with the power of 300 W and found that a process time between 20 and 35 min allowed the sacrificial to be completely removed without any cap damage. Otherwise, for a time longer than 35 min, caps partially collapsed on the CPW and were completely down under the application of a stylus tip force as low as 20 µN. A time of 80 min was required for complete release when the low powers of 200 W or 100 W were used.

## 6. RF Modeling 

A full 3D FEM model was developed to simulate the RF behavior of 400 µm long CPWs in gold on low (ρ = 20 Ohm·cm) and high (ρ = 5 kOhm·cm) resistivity silicon substrates. Simulations of CPWs uncapped and encapsulated with the fabricated caps having A = 26%, 50%, and 57% were performed in the frequency range of 0–40 GHz.

**Figure 4:**
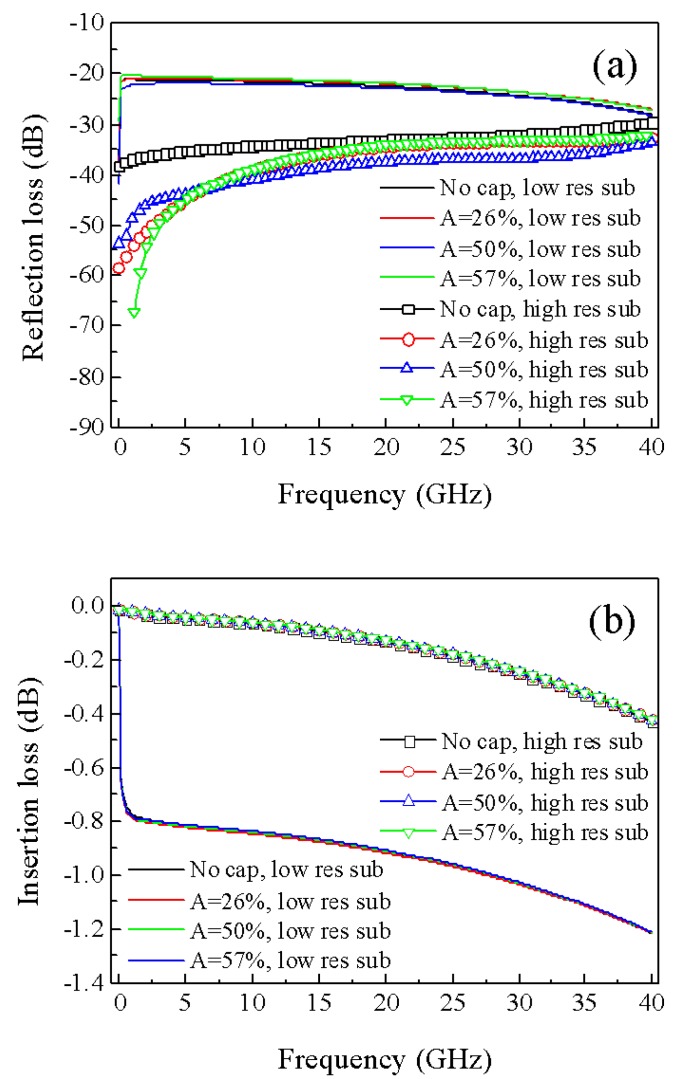
Magnitude of simulated reflection (**a**) and insertion (**b**) losses for gold coplanar waveguides (CPWs) uncapped and encapsulated with the fabricated caps having A = 26%, 50%, and 57% on low and high resistivity silicon substrates.

For low resistivity substrate, the reflection loss was predicted to decrease with increasing the frequency from around −21 dB to −28 dB (Figure 4a) for uncapped and capped CPWs. No significant variation was observed as a function of the cap, likely due to the fact that substrate losses and characteristic impedance mismatch between CPW and RF terminations (i.e., input/output ports in the simulated model) dominate. 

For a high resistivity substrate, the reflection loss of the uncapped CPW was significantly smaller than that on the low resistivity substrate, due to a better match of the CPW characteristic impedance, with values that increased with frequency from around −38 dB to −30 dB. In the presence of a cap, the reflection loss of the CPW on the high resistivity substrate further decreased, especially for frequencies lower than 6 GHz. In particular, the reflection loss reduced by a factor of ~0.87 for almost all frequencies for the capped CPW with A = 50% and decreased for frequencies lower than 15 GHz by a factor up to 1.8 for capped CPWs with A = 26% and 57%. The reduction of reflection loss observed in the presence of a cap can be ascribed to the fact that the CPW geometry was not previously optimized for any specific frequency so that it is possible that the CPW characteristic impedance gets closer to 50 Ohm thanks to the interaction of the electromagnetic field with the cap.

The insertion loss of the uncapped CPW on a low resistivity substrate decreased from around −0.8 dB to −1.2 dB with frequency (Figure 4b). When the high resistivity substrate was used, the insertion loss of the uncapped CPW improved, reducing from nearly zero to around −0.45 dB. For both substrates, no appreciable effects on the insertion loss were predicted to be induced by the presence of caps.

## 7. Conclusions

A fabrication process of thin film encapsulating caps on CPWs was developed. The tensile stress of deposited SiN_x_/a-Si/SiN_x_ trilayers gave a concave shape to the caps and made them robust and flexible without breakage under the pressure of a profiler stylus tip, even with a percentage of the perforated cap area as high as 74%. Two etching processes were performed in the same barrel chamber both to create a matrix of holes with a diameter of 7 µm and 8 µm through the caps, and to remove the sacrificial layer under them. The removal of the sacrificial layer was investigated as a function of the percentage of the cap perforated area for etching times and powers in the ranges of 8–80 min and 100–600 W, respectively. The widening of etch holes, due to the isotropic nature of the barrel etching, will be taken into account in the design of the next caps, in order to achieve holes with a size favorable for cap sealing. Future work will be focused on the sealing process; the mechanical robustness of the fabricated encapsulating thin films under the external pressure load as well as their reliability in dusty or high humidity environments will be evaluated, as required in some real applications. For a gold CPW on a high resistivity silicon substrate, the fabricated caps were predicted to reduce the reflection loss by a factor up to 1.8 and not degrade the insertion loss. 

The oxygen plasma-based release of caps and the reasonably low temperatures (<200 °C) of all involved processes make the fabricated thin film encapsulation caps compatible with a wide range of microsystem technologies, from surface to high aspect ratio microstructures, also integrated with CMOS devices. In particular, the low impact of the fabricated caps on RF performance of gold CPWs is a good premise for transferring this packaging solution to RF MEMS switches, switching units, and other complex devices, such as reconfigurable phase shifters, tunable filters, and digital step attenuators, for 5G and modern telecommunication systems applications. 

## Figures and Tables

**Table 1:** Parameters of barrel etching processes for cap patterning and release.

	Chamber Pressure (mTorr)	O_2_ Flow (sccm)	SF_6_ Flow (sccm)	Ar flow (sccm)	P (W)	t (min)
Patterning SiN_x_	200	10	90	/	200	1–1.5
Patterning a-Si	400	10	90	15	200	2
Release	600	300	3	/	100–600	8–80

**Table 2:** Number of holes with diameter (D) of 7 and 8 µm in the various fabricated caps and resulting percentage of perforated area.

	N	A
D = 8 µm	165	26%
	320	50%
	480	74%
D = 7 µm	480	57%

**Table 3:** Barrel parameters for the etching of the sacrificial layer and consequent results in terms of release for capping layers with A = 57%.

	Barrel Parameters	Cap Release
Test 1	P = 600 W, t = 8 min	complete
Test 2	P = 400 W, t = 8 min	complete
Test 3	P = 300 W, t = 8 min	incomplete
Test 4	P = 300 W, t = 20 min	complete
Test 5	P = 200 W, t = 20 min	incomplete
Test 6	P = 200 W, t = 80 min	complete
Test 7	P = 100 W, t = 80 min	complete
